# Comparative Assessment of the Accuracy of Digital Versus Conventional Interocclusal Records in Dentulous Patients: A Systematic Review

**DOI:** 10.7759/cureus.72002

**Published:** 2024-10-21

**Authors:** Divya Nagri, Neha Jain, Pankaj Dhawan

**Affiliations:** 1 Department of Prosthodontics, Manav Rachna Dental College, Faridabad, IND; 2 Department of Prosthodontics and Crown and Bridge, Manav Rachna Dental College, Faridabad, IND

**Keywords:** accuracy, conventional, digital, interocclusal records, intraoral scanner, occlusal registration material, virtual

## Abstract

Interocclusal registration is an essential step in oral rehabilitation. Conventionally, interocclusal registration has been conducted using interocclusal records made of wax or silicone. However, a digital method of registration utilizing an intraoral scanner has been introduced for analyzing inter-arch relationships. This systematic review aims to compare digital interocclusal records with conventional interocclusal records by considering their respective quality, such as accuracy. A thorough search was conducted to gather relevant research from databases such as Google Scholar, PubMed, and Scopus. After evaluating the study's methodological quality assessment using NIH's Quality Assessment Tool for Observational Cohort and Cross-Sectional Studies, two independent reviewers assessed the publications for inclusion and exclusion criteria. After a methodical search, a total of five relevant papers were reviewed. The review's primary finding was that digital interocclusal records were more accurate than conventional interocclusal records.

## Introduction and background

Establishing a functional occlusion is primary goal in restorative dentistry for all prosthodontists. Premature contacts, interferences of cusps, and over or under-reduction in tooth preparations are a few reasons for the failure of the restorations. The tooth reduction and occlusion establishment being very critical, different materials and techniques have been clinically tried for establishing harmonious functional occlusion. Traditionally, interocclusal records have been the gold standard for recording maxillo-mandibular relationships. In the modern era of dentistry, digital interocclusal recordings are made possible by examining the labial and buccal surfaces of teeth when they are in the maximum intercuspation position. Although these buccal-occlusal (virtual) images are frequently utilised to create restorations, there is little proof that the procedure is clinically accurate. As a result, in order to support evidence-based clinical decision-making, all dentists must be aware of this latest development and the significance of their comparative study [1-2].

Review scope

This systematic review will focus on the assessment of the accuracy of digital interocclusal records in comparison with conventional interocclusal records. In clinical practice, traditional occlusal registration procedures are being replaced by digital methods. In light of the dearth of research evaluating the clinical accuracy of virtual occlusal registration, this systematic review aims to assist clinicians in choosing the best technique for creating interocclusal records, which will eventually allow a patient to have an indirectly manufactured prosthesis placed in their mouth without experiencing occlusal errors [3-7].

Objective

This study's primary goal is to compare and assess the accuracy of the digital interocclusal record in comparison with the conventional interocclusal record. Our focus will be on carefully examining the existing evidence and offering insights that should assist clinicians in selecting the most accurate interocclusal record method for bite registration. This will enable the clinician to provide patients with a better prosthesis while minimizing chair-side occlusal adjustment.

## Review

Materials and methods

**Figure 1:**
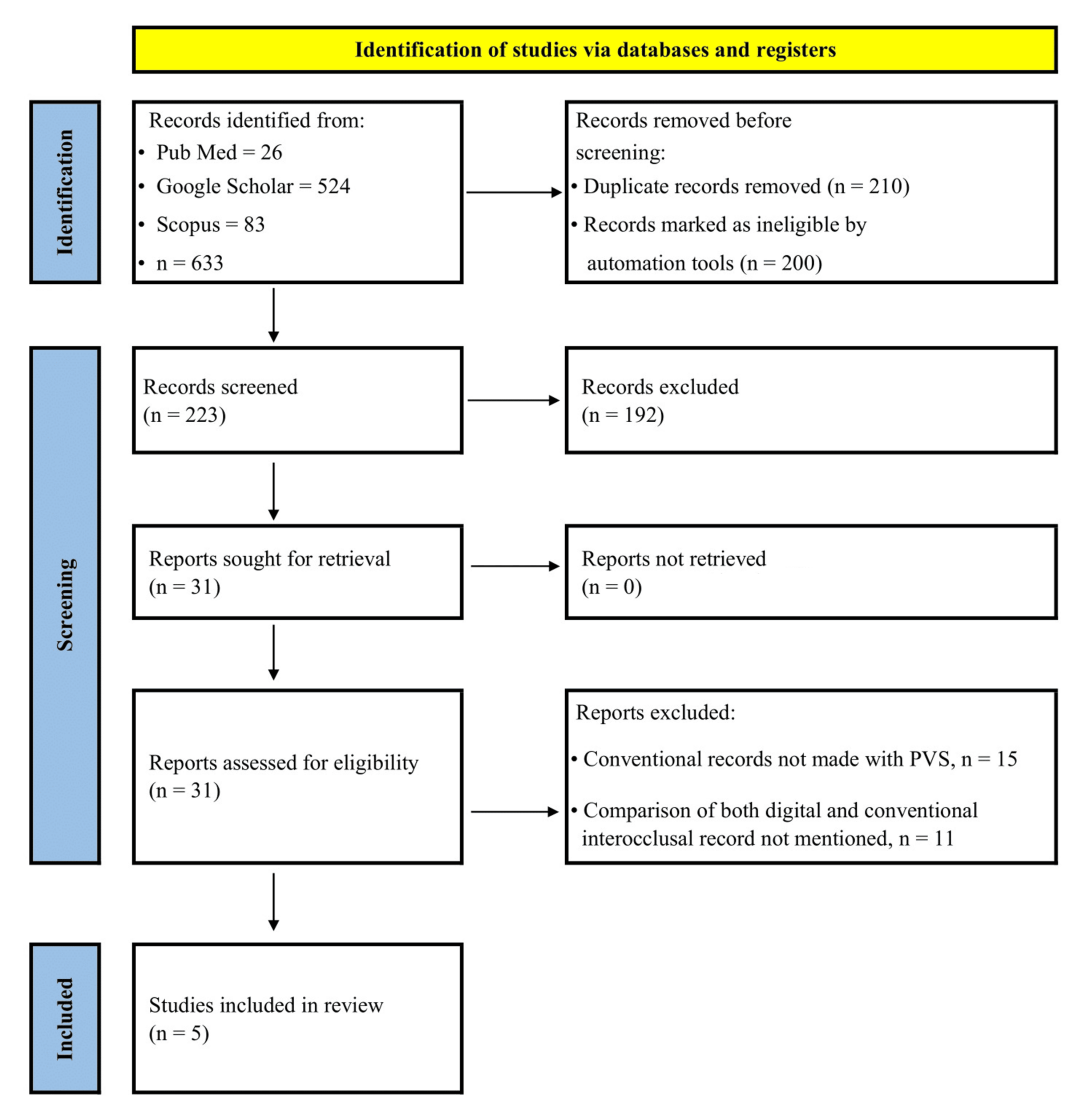
PRISMA flow chart

This systematic review was conducted using the Preferred Reporting Items for Systematic Review and Meta-Analysis (PRISMA) standards (Figure 1). PROSPERO (the International Prospective Register of Systematic Review, CRD420245656979580) is where the protocol is registered. A search was conducted on Medline (PUBMED), Scopus, and Google Scholar with the following category-grouped search items: the category-grouped search items include adults with teeth as the patient population, digital interocclusal records as the intervention, conventional silicone interocclusal records as the control, and the accuracy of interocclusal records as the result.

Search strategy

A systematic search was performed on Scopus, Google Scholar, and PubMed databases from 2015 to 2024. Abstracts were also assessed when possible. Every database had a unique set of electronic search tactics. The search technique only allowed for English language use. Terms and Keywords from the Medical Subject Headings (Mesh) were matched in pairs.

The following search strategy was used: digital interocclusal record* OR optical interocclusal record* OR virtual interocclusal record* OR intraoral scanner*OR 3dimensional scanner* AND conventional interocclusal record with polyvinyl siloxane AND accuracy OR precision.

Inclusion Criteria

RCT, clinical trials and cross-sectional studies, studies in which the accuracy of the digital interocclusal record was examined, studies in which the accuracy of interocclusal record via silicones was evaluated, papers written in English or in other languages for which an English translation is feasible.

Exclusion Criteria

Reviews, analyses by specialists, case studies, abstracts, and research on permanent teeth were all disqualified.

Systematic Search Strategy

To find pertinent studies, two impartial reviewers carefully carried out a three-step selection procedure. The first step was a title scan, in which papers that weren't relevant were disregarded after a brief title review. The second phase involved conducting an abstract and summary drive, wherein abstracts and summaries were scrutinised to select studies according to several criteria such as patient population size, research methodology, digital intervention kind, and outcome variables evaluated. The complete texts of the studies that made the shortlist were carefully examined during the third step of data extraction.

Data Extraction

Three reviewers (DN, NJ, and PD) carried out this systematic review. The writer DN carried out the main database search for relevant literature and separately reviewed the titles for potential abstract amendment. Based on inclusion and exclusion criteria, the papers underwent additional screening. For the papers that satisfied the inclusion requirements, full texts were acquired, and NJ assessed the articles' bias before conducting additional review work. The author created the data extraction spreadsheet and filled it with the following details: author, publication year, study design, workflow or restoration type, scanner type, study goal, accuracy measurement technique, and study outcome in terms of accuracy or precision. PD was consulted in case of ambiguity.

Risk of bias and quality assessment

Two impartial reviewers evaluated the included studies' quality and bias risk. After removing redundant sources, study titles and abstracts were examined to make sure they were pertinent. Observational studies were critically evaluated using an NIH methodology.

Bias Assessment Within Individual Studies

The risk of bias as described in Table 1 was determined depending on the quality of the article by utilizing NIH's Quality Assessment Tool for Observational Cohort and Cross-Sectional Studies.

Quality was rated as poor for a score of 0-4 out of 14 questions, fair for 5-10 out of 14 questions, and good for 11-14 out of 14 questions. If the article had good quality, then it meant low risk of bias. Quality was rated as follows: 0 for poor (0-4 out of 14 questions), i for fair (5-10 out of 14 questions), and ii for good (11-14 out of 14 questions). NA indicates 'not applicable,' while NR means 'not reported.

**Table 1:** Bias assessment within individual studies

Variables	Abdulateef et al. [8] (2019)	Ries et al. [10] (2021)	Iwauchi et al.[9] (2021)	Morsy et al.[11] (2022)	Das et al. [12] (2022)
Was the research question or paper clearly stated?	Yes	Yes	Yes	Yes	Yes
Was the study population clearly specified and defined?	Yes	Not Applicable	Yes	Yes	Yes
Was the participation rate of eligible persons at least 50%?	Yes	Not Applicable	Yes	No	Yes
Were all the subjects selected or recruited from the same or similar population? Were the inclusion and exclusion criteria for being in the study prespecified and applied uniformly to all participants?	Not Applicable	Not Applicable	Not Relevant	Yes	Cannot Determine
Was a sample size justification, power description, or variation and effect estimates provided?	Not Applicable	No	Not Reported	Yes	Yes
For the analysis in this paper, was the exposure of interest measured prior to the outcome being measured?	Yes	Yes	Yes	Yes	Yes
Was the time frame sufficient so that one could reasonably expect to see an association between exposure and outcome if it existed?	Yes	Yes	Yes	Yes	Yes
For exposure that can vary in amount or level, did the study examine different levels of exposure?	Yes	Yes	Yes	Yes	Yes
Were the exposure measures clearly defined, valid, reliable, and implemented consistently across the study?	Yes	Yes	Yes	Yes	Yes
Was the exposure assessed more than once over time?	Yes	Yes	Yes	Yes	Yes
Were outcome assessors blinded to the exposure status of participants?	No	Not Applicable	Yes	No	No
Was loss to follow-up after baseline 20% or less?	Not relevant	Not relevant	Yes	Not relevant	Cannot determine.
Were key potential confounding variables measured and adjusted statistically for their impact on the relationship between exposure and outcome?	Yes	Not Applicable	Yes	Yes	No
Quality of the article:	Fair	Fair	Good	Fair	Fair

Results

As outlined in the PRISMA flow chart, studies that remained after title and abstract screening were evaluated for eligibility. A total of five studies were included in the literature of this systematic review. The details of the studies reviewed are described in Table 2.

**Table 2:** Narrative synthesis has been provided for the findings obtained from the studies. The data extracted has been presented in the tabular form N: Number (of participants); PVS: polyvinyl siloxane; STL: Standard Tessellation Language; IOS: intraoral scanner

Year	Author	Study Design	Type of workflow or restoration	Type of scanner used	Objective	Method of measuring the agency	Results (p< 500um)
2019	Abdulateef et al. [8]	Clinical trial	N=10, Group 1: Unilateral interocclusal record on the right and left side with PVS; Group 2: Virtual interocclusal records. An independent software program detected the site of close proximity and the site of clearing on both the transilluminated conventional PVS record and the virtual record.	CEREC Omnicam (Dentsply Sirona Corp., Charlotte, NC)	Accuracy of interocclusal record	A comparison software program was used to analyse STL files and measure the "nearest neighbour" distances between the mesh faces and vertices of opposing arches.	Clinically acceptable levels of accuracy are achieved in recognising interoocclusal interactions using virtual interocclusal data.
2021	Iwauchi et al. [9]	Clinical trial	N=8; Group 1: Digital scan with True definition IOS; Group 2: Digital scan with TRIOS IOS; Group 3: Conventional Interocclusal record with silicone.	True Definition IOS (3M, St. Paul, MN); TRIOS IOS, 3Shape, Copenhagen, Denmark.	Precision of interocclusal record	STL files were analysed by using the best-fit algorithm	Intermaxillary relationships captured by digital methods had better precision than conventional methods.
2021	Ries et al. [10]	Clinical trial	N=15; Group 1: Digital interocclusal record; Group 2: Conventional interocclusal record with PVS	TRIOS IOS	Accuracy of interocclusal record	STL files were analysed by using the best-fit algorithm.	Digital interocclusal registrations’ showed greater accuracy in comparison with conventional interocclusal registration method.
2022	Morsy et al. [11]	Randomized control clinical trial	N=9; Group 1: Full arch digital impression and virtual interocclusal record. Group 2: Quadrant arch digital impression and virtual interocclusal record. Group 3: Full arch conventional impression and silicone interocclusal record. Group 4: Quadrant arch conventional impression and silicone interocclusal record	Medit i700 IOS(Medit, Seoul, South Korea)	Precision of interocclusal record	STL files were analysed by using the best-fit algorithm	In entire and quadrant arch scenarios, the digital approach greatly outperformed the conventional method in terms of precision for static interocclusal registration.
2022	Das et al. [12]	Clinical trial	N=8, with complete natural dentition. Inter occlusal registration in Group 1 with 3M True Definition Scanner; Group 2: Intraoral scanning with Trios Scanner 3; Group 3: Conventional interocclusal record by silicone, scanned by the 3D laboratory scanner.	True Definition IOS and TRIOS Scanner 3	Precision of interocclusal record	STL files were analysed by using the best-fit algorithm	The accuracy of the intermaxillary connection established by the IOS digital scan method was superior to that of the conventional method.

Discussion

This systematic review's goal was to compare and assess the accuracy of digital interocclusal records in comparison with conventional interocclusal records. Five articles were included in the study out of which one was a randomized control trial and the rest were clinical trials. The outcome of this systematic review showed that when compared to conventional interocclusal records made with polyvinylsiloxane, the accuracy of digital interocclusal records is higher. Trueness and precision are the metrics used by the International Organization for Standardization to assess accuracy. Trueness is the measuring bias or systematic error between the referred object and the target object. High trueness yields a result that approaches or matches the target object's and the measured object's real dimension. On the other hand, a higher precision denotes more consistent measurements. When an operation is repeated, the random error over all objects serves as a measure of precision. These clinical studies solely assessed the interocclusal registration's precision because a clinical evaluation of trueness is not feasible due to a lack of standard data [13-15].

The results of this systematic study corroborate reports from Iwauchi et al. [9] and Ries et al. [10], according to which the virtual interocclusal record is a more accurate method than the conventional method when using polyvinylsiloxane. The review's findings concur with Camcı et al [16]. Additionally, Ayuso-Montero et al. [17] found that virtual interocclusal records using intraoral scanning had superior precision than conventional interocclusal records in identifying the occlusal contact area. However, the result of this study disagrees with Abdulateef et al. [8] which stated that it is therapeutically acceptable for virtual interocclusal records to accurately detect interocclusal interactions; however, the intraoral system tends to miss interocclusal contacts rather than introduce false contacts. The heterogeneity inherent in clinical investigations may be the cause of the variations in the results, as it can be challenging to standardize participant jaw motions, occlusal forces, and dental occlusion.

The software best-fit-alignment algorithm was used in these studies to assess precision. In order to measure precision, Standard Tessellation Language (STL) files from digital and conventional interocclusal records (achieved by laboratory scanning of the articulated cast) were superimposed on one another. The Hausdorff distance is defined as the maximum distance in the x, y, and z axes between any point on one STL file and its nearest point on another STL file [11].

According to Morsy et al. [11], when stone castings are mounted using a silicone bite, improper seating of the stone casts in the bite, dimensional changes in the silicone, and compressibility of the silicone bite can all lead to poor articulation. Moreover, when the indirect digital workflow is used, mistakes could happen when scanning mounted castings with silicone bites, which are scanned in order to create virtual articulation from actual articulation. The mounted castings, which are larger and heavier than single casts, are rotated, translated, and tilted by the laboratory scanner as it scans. This movement may cause the articulated pair of casts to shift and alter the patient's recorded maximum intercuspation position record. The ABO objective grading system states that a precision mean value of less than 500um was deemed sufficient for this inquiry. As the fixed prosthodontics articulation tolerance, a deviation in cast articulation of up to 500um might be regarded as significant [11].

Digital interocclusal records have shown significant promise in improving accuracy. Intraoral scanners and digital bite registration systems often provide more precise and reproducible results due to their ability to capture detailed 3D data without the limitations inherent in material-based records. Several studies have demonstrated that digital records can achieve higher accuracy in terms of occlusal fit and spatial relationships, potentially reducing adjustments required during prosthetic delivery. However, Patil et al. [18] evaluated the bite registration accuracies with intraoral scanning and determined that the registration of digital bites is better than conventional bite registration for quadrant scan as compared to complete arch scan. They also concluded that the digital method has certain limitations like differences in the scanning technology and virtual articulation algorithm could lead to potential distortions in digital interocclusal data.

This systematic evaluation was conducted with the intention of assisting practitioners in making therapeutic decisions regarding the adoption of digital bite records vs conventional ones. The review's findings supported employing digital bite registration to provide virtual articulation that is more accurate. One of the study's limitations was that accuracy was evaluated solely on precision. However, trueness was not possible to be assessed in this review as the clinical research included was in vivo, where it was not possible to establish a reference standard. It is advised to conduct additional research to evaluate the reproducibility of various intraoral scanners for virtual interocclusal records. The five publications that we referred to for systematic analysis included four that produced statistical analysis results and shared the same approach, but one of the articles included an in vitro study, which suggests that the articles were biased toward heterogeneous data. There was no meta-analysis done in this systematic review because of the bias risk, poor methodological quality, and heterogeneity of the data.

## Conclusions

This systematic review concluded that digital scan techniques and intraoral scanners have more accuracy than those obtained by conventional ways that used silicone impression material and a gypsum cast. The accuracy of the intraoral scan and virtual interocclusal record is better for the quadrant arch as compared to the complete arch. The interocclusal record obtained by a completely digital workflow is more accurate than the digitization of conventional interocclusal records. It could also be concluded that intraoral scanning systems tend to miss interocclusal contacts however do not introduce false contacts. It could also be stated that clinicians should be careful with the type of intraoral scanning used as they can result in inherent distortions in digital interocclusal records. Also, the algorithm used to superimpose can lead to variation in the precision of the interocclusal record obtained.
